# Imaging in Van Wyk Grumbach syndrome: An uncommon presentation of hypothyroidism

**DOI:** 10.4102/sajr.v27i1.2572

**Published:** 2023-03-15

**Authors:** Poonam Sherwani, Khanak K. Nandolia, Kirti Joshi, Radhapyari Lourembam

**Affiliations:** 1 Department of Radiodiagnosis, All India Institute of Medical Sciences Rishikesh, Rishikesh, India; 2 Department of Paediatrics, All India Institute of Medical Sciences Rishikesh, Rishikesh, India

**Keywords:** galactorrhoea, hypothyroidism, isosexual precocious puberty, pituitary, ovarian mass, thyroid stimulating hormone (TSH), per vagina, Van Wyk Grumbach syndrome (VWGS)

## Abstract

Isosexual precocious puberty with ovarian masses in long-standing juvenile hypothyroidism is well described in the literature as Van Wyk Grumbach syndrome (VWGS). The present case reports this rare entity in a 4-year-old girl who was referred for imaging to evaluate the cause of non-traumatic bleeding per vagina. Antecedent history, clinical features and thyroid function tests were consistent with long-standing juvenile hypothyroidism with documented clinical response to thyroxine replacement therapy.

**Contribution:**

Typical clinical and radiological features of the syndrome are reported, which helps in the early diagnosis and management, henceforth avoiding the associated complications.

## Introduction

Van Wyk Grumbach syndrome (VWGS) is a rare presentation of untreated hypothyroidism which is the most commonly encountered endocrine disorder in children. Classic clinical features of VWGS are precocious puberty, galactorrhoea and cystic ovarian lesions that are remarkably extenuated following thyroxine replacement therapy.^1^ Precocious puberty is due to high levels of thyroid stimulating hormone (TSH) causing stimulation of the follicle-stimulating hormone (FSH) receptors. Pituitary hyperplasia is also often seen in VWGS. Comprehensive knowledge about imaging in VWGS is imperative for radiologists who can advise against unnecessary surgical interventions.

## Case Report

A 4-year-old girl presented to the Emergency Department of a tertiary care institute with heavy fresh, red-coloured bleeding per vagina in the absence of trauma or sexual assault. Short stature, short and stubby hands, delayed dentition, bradycardia and various other general features on physical examination were suggestive of hypothyroidism. The thyroid gland was not palpable. Haematological investigations revealed macrocytic anaemia and a normal coagulation profile. Thyroid profile showed elevated levels of TSH (699 IU/mL, normal range 0.35 IU/mL – 4.9 IU/mL) with low levels of serum T3 (< 1 pg/mL, normal range 2.6 pg/mL – 4.8 pg/mL) and serum T4 (< 0.4 ng/dL) along with a markedly elevated level of Thyroid Peroxidase Oxidase (2152 U/mL normal range 0–60). Basal FSH was elevated; however, Luteinizing hormone (LH) and Prolactin levels were normal. Based on the biochemical findings, a diagnosis of autoimmune hypothyroidism was made.

**FIGURE 1:**
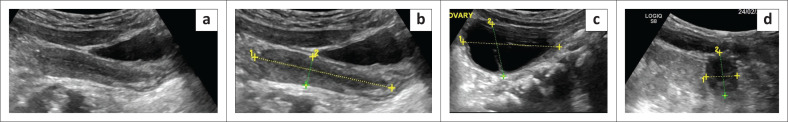
(a-b) Pelvic ultrasound in the sagittal plane depicting an enlarged uterus for a 4-year-old girl with a trilaminar endometrium similar to the post-pubertal uterus. (c) Septated cystic lesion in the right adnexa. (d) The left ovary is bulky with a dominant follicle.

Greyscale ultrasonography and colour flow Doppler imaging of the pelvis revealed a post-pubertal sized uterus, measuring 6.3 cm × 1.3 cm (length × transverse) with a tri-layered endometrium. A septated cystic lesion, measuring 5.7 cm × 3.7 cm, was seen in the right adnexa with no peripheral or septal colour flow. The right ovary was not separately visualised. A dominant follicle was seen in the bulky left ovary (Figure 1).

**FIGURE 2:**
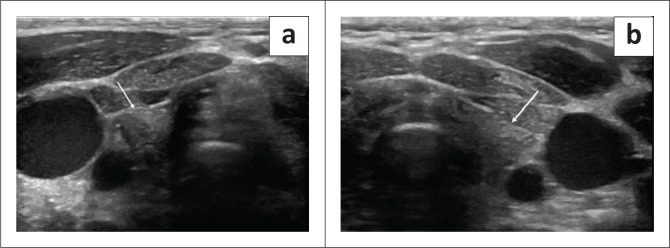
Axial ultrasound images of the thyroid gland showing a hypoplastic right lobe (arrow in a) and left lobe (arrow in b). The isthmus is not visible in the midline.

Ultrasonography of the neck revealed bilateral hypoplastic thyroid lobes in the normal position. The right thyroid lobe measured 6.5 mm × 5.5 mm and the left lobe measured 8.8 mm × 6 mm. The isthmus was not visualised. There were no thyroid nodules or cysts (Figure 2).

**FIGURE 3:**
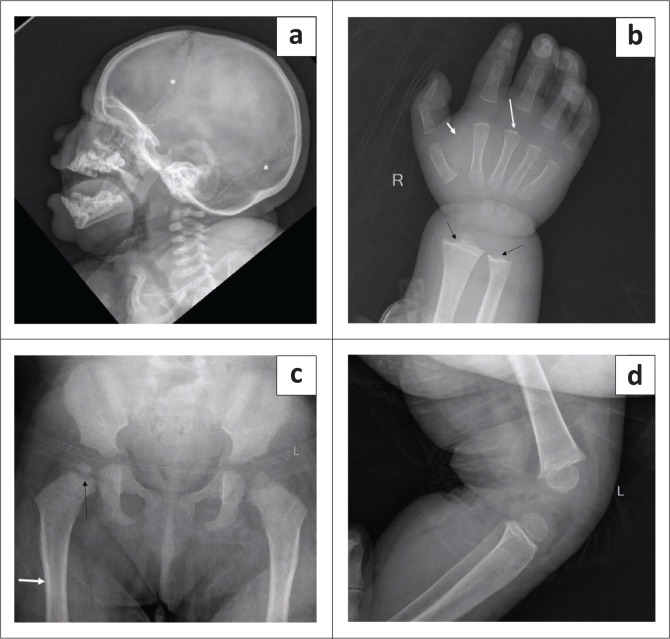
Lateral radiograph of the skull (a) showing multiple Wormian bones in the coronal and lambdoid sutures (*). Frontal radiograph of the right wrist (b) showing a delayed appearance of ossification centres for the carpal bones. Bone age corresponded to more than 1 year and less than 4 years. Metacarpal epiphyseal ends show a small spike-like projection extending into the growth plate (long white arrow). Irregularity of the growth plate is seen along the distal end of the radius and ulna (black arrows) and there is soft tissue hypertrophy (small white arrow). (c) Frontal radiograph of the pelvis and proximal femurs shows fragmented epiphysis (black arrow) with cortical thickening of both proximal femoral diaphysis (arrow in c). (d) The lateral radiograph of the left knee is unremarkable.

**FIGURE 4:**
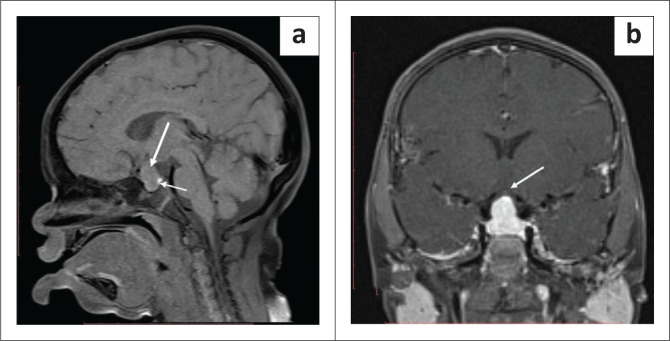
(a) Sagittal T1W images show a T1 iso-intense sellar and suprasellar mass (long white arrow). The posterior pituitary is seen separate from the mass as a T1 bright spot (small white arrow in a). (b) Gadolinium-enhanced coronal images demonstrate the homogeneous enhancement of the mass with elevation of the optic chiasm anteriosuperiorly (arrow in b).

A skeletal survey was performed to assess bone age. A wrist radiograph revealed ossification centres for the hamate and capitate, consistent with a bone age of 3–4 months. The appearance of the epiphysis at the distal end of the radius was consistent with a bone age of 1 year. Triquetral and lunate bone ossification centres were not seen indicating that the bone age of the child corresponded to more than 1 year but less than 4 years (Figure 3b).^2^ A lateral radiograph of the skull revealed the presence of multiple Wormian bones and an enlarged sella (Figure 3a). Radiographs of the pelvis and hip joints exhibited diffuse cortical thickening along the meta-diaphyseal region of both proximal femora (Figure 3c). The lateral radiograph of the left knee was unremarkable (Figure 3d). Contrast-enhanced magnetic resonance imaging for the pituitary gland revealed hyperplasia of the anterior pituitary with homogeneous enhancement. The posterior pituitary was unremarkable (Figure 4).

Most of the causes of precocious puberty are associated with advanced bone age except for VWGS where delayed bone age is seen which leans towards the diagnosis.^3,4^ Therefore, based on the clinical, biochemical, and radiological findings, a diagnosis of VWGS was made and the child was treated with thyroxine. Five days after initiation of therapy, the vaginal bleeding stopped and the child was discharged with recommendation for follow-up.

**FIGURE 5:**
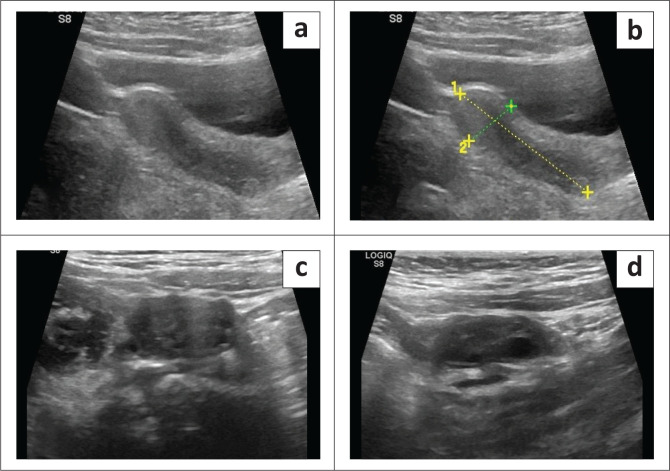
(a-d) A follow-up ultrasound after 2 months showed a significant reduction in the size of the uterus and regression in the size of both ovaries, with almost complete resolution of the cysts.

At follow-up after 2 months, the child showed significant improvement in clinical symptoms. There was a substantial reduction in weight, with marked improvement in appetite and constipation. Facial features also showed marked improvement. Ultrasound pelvis revealed a significant decrease in the size of the uterus, which measured 3.8 cm × 1.3 cm. Regression in the size of the ovaries was seen with the complete resolution of the cyst and follicle. The right ovary measured 2.2 cm × 1.5 cm and the left ovary measured 2 cm × 0.8 cm. (Figure 5)

## Discussion

Van Wyk and Grumbach described the syndrome of precocious puberty, galactorrhoea, and bilateral ovarian masses in primary hypothyroidism with complete reversal of the symptoms after thyroid replacement in 1960.^5^ Bilateral ovarian masses and precocious puberty suggest the diagnosis of an oestrogen secreting neoplasm. A possible diagnosis of VWGS should be considered when bilateral cystic ovarian masses are associated with long-standing hypothyroidism, and long-term complications can be avoided by the timely initiation of thyroid replacement therapy.^6^ Patients usually present with typical clinical features of hypothyroidism which include weight gain, poor concentration, depression, fatigue and menstrual irregularities. The index case also presented with thelarche, early menarche and precocious puberty. Most of the cases are reported in females^7^ and very few cases have been reported in boys.^8^ Long-standing hypothyroidism, high levels of TSH, precocious isosexual puberty and delayed bone age are the pertinent features of this syndrome which were seen in the presented case.^1^ Bone changes in hypothyroidism generally include delayed bone age, sellar enlargement secondary to pituitary hyperplasia, multiple Wormian bones, and hypoplastic sinuses.

Childhood hypothyroidism can be either congenital or acquired, primary or secondary, or due to central or peripheral causes. Primary hypothyroidism is due to abnormality at the level of thyroid, which includes either defective thyroid gland development, termed dysgenesis, or thyroid hormone dysgenesis known as dyshormonogenesis. Secondary hypothyroidism is due to deficiency of TSH, which can be either isolated or seen in panhypopituitarism. Peripheral hypothyroidism is due to a defect in transport, metabolism or action. In the presented case, hypothyroidism was due to autoimmune thyroiditis, the most common cause of acquired hypothyroidism in children and adolescents and is most commonly associated with the syndrome.^9^ Hypothyroidism can be either permanent or transient. Transient hypothyroidism is either due to iodine deficiency or maternal blocking antibodies.^10^

**FIGURE 6:**
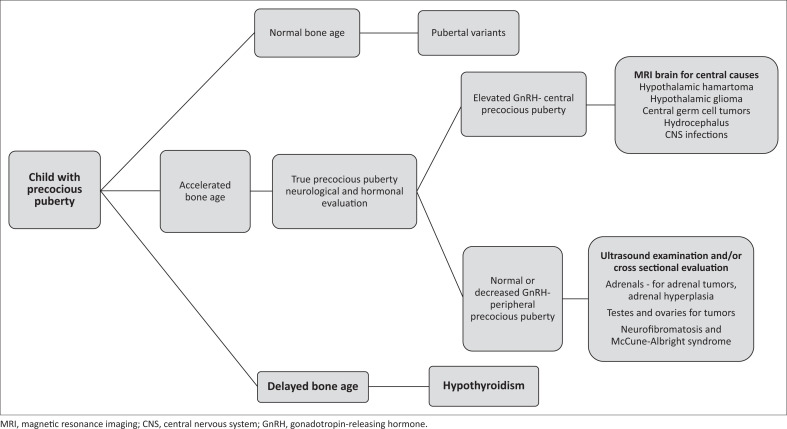
Flow chart depicting an algorithm for the radiological approach to a child with precocious puberty.

Various theories have been postulated regarding precocious puberty in congenital hypothyroidism. The proposed mechanism by Van Wyk and Grumbach was high levels of TSH, leading to the elevated levels of gonadotrophins through the pituitary hypothalamic axis. However, some studies have also postulated that there is no elevation of gonadotrophins from the pituitary. Instead, there is stimulation of the Follicle-stimulating hormone receptor and not the Leutinizing hormone receptor, which accounts for isosexual precocious puberty in these cases, which causes multicystic ovaries, uterine enlargement, bleeding per vagina and thelarche. Precocious puberty is disconsonant in these cases as there is no stimulation of adrenarche, and axillary and pubic hair growth does not occur. Another theory of prolactin relates well with the discordance of FSH and LH. Due to the unopposed action of thyrotropin-releasing hormone (TRH), there is hyperprolactinaemia which causes increased ovarian sensitivity to gonadotrophins which in turn causes a slow release of gonadotropin-releasing hormone, which suppresses LH while producing FSH.^4^ Myxoedematous infiltration of the ovary also accounts for its enlargement.^11^ Continuous TRH leading to increased FSH secretion is also one of the proposed theories for precocious puberty.^12^ An algorithmic approach for the child with precocious puberty is presented for the radiologist in the flow chart (Figure 6).^4^

Precocious puberty in VWGS presents with unique features of delayed bone age as well as short stature, which differs from other causes of precocious puberty where growth acceleration is the criteria as the thyroid hormone causes bone maturation directly through T3 and indirectly through the growth hormone regulation gene.^13^ Long-standing hypothyroidism leads to thyrotroph hyperplasia leading to pituitary hyperplasia. Pituitary macroadenoma remains a close differential diagnosis,^14^ although macroadenoma is rare in children. Imaging features such as homogenous enhancement, midline location with a smooth bulge and the presence of posterior bright spot favour hyperplasia. In contrast, heterogeneous enhancement, off midline location, and the absence of pituitary bright spot in up to 20%, favour macroadenoma.^14^

## Conclusion

The triad of precocious puberty, delayed bone age, and bilateral cystic ovarian lesions with features of hypothyroidism suggest the diagnosis of VWGS. Early recognition of symptoms and treatment with thyroxine show clinical and radiological improvement and also avert the delayed complications associated with hypothyroidism.

## Learning Points

- Precocious puberty, delayed bone age and cystic ovarian masses in long-standing juvenile hypothyroidism propose the diagnosis of VWGS.
- Various clinical features seen in VWGS syndrome resolve with thyroxine replacement therapy and once diagnosed, medical treatment can be initiated early by the referring doctor.
- Early recognition can avoid the delayed complications of the syndrome which include short stature and psychosocial impairment.
